# A severe case of human coronavirus 229E pneumonia in an elderly man with diabetes mellitus: a case report

**DOI:** 10.1186/s12879-021-06188-3

**Published:** 2021-06-04

**Authors:** Wen Sun, Ji-Ping Liao, Kun-Yao Yu, Jian-Xing Qiu, Chen-Li Que, Guang-Fa Wang, Jing Ma

**Affiliations:** 1 Department of Pulmonary and Critical Care Medicine, Peking University First Hospital, Beijing, 100034 China; 2 Department of Radiology, Peking University First Hospital, Beijing, 100034 China

**Keywords:** Adult respiratory distress syndrome, Human coronavirus 229E, Immunology, Pneumonia

## Abstract

**Background:**

With pandemic of coronavirus disease 2019 (COVID-19), human coronaviruses (HCoVs) have recently attached worldwide attention as essential pathogens in respiratory infection. HCoV-229E has been described as a rare cause of lower respiratory infection in immunocompetent adults.

**Case presentation:**

We reported a 72-year-old man infected by HCoV-229E with rapid progression to acute respiratory distress syndrome, in conjunction with new onset atrial fibrillation, intensive care unit acquired weakness, and recurrent hospital acquired pneumonia. Clinical and radiological data were continuously collected. The absolute number of peripheral T cells and the level of complement components diminished initially and recovered after 2 months. The patient was successfully treated under intensive support care and discharged from the hospital after 3 months and followed.

**Conclusion:**

HCoV-229E might an essential causative agent of pulmonary inflammation and extensive lung damage. Supportive treatment was essential to HCoVs infection on account of a long duration of immunological recovery in critical HCoV-229E infection.

## Background

Human coronaviruses (HCoVs) are enveloped, single-stranded RNA viruses [1]. HCoV-229E, as a cause of the common cold [1], rarely leads to lower respiratory infection in immunocompetent adults. Recently, HCoV infections have attracted the world’s attention due to the coronavirus disease 2019 (COVID-19) pandemic [2].

Herein, we report a case of severe pneumonia caused by HCoV-229E with continuous monitoring of the systemic immune response, and review the literature on HCoV strains.

## Case presentation

A 72-year-old man went to the emergency department (day 3) on Nov 4, 2019, with a 3-days history of fever (38.3 °C/100.94 °F), fatigue, myalgia and nonproductive cough, and a 1-day history of dyspnea. In the past 30 years, the patient had diabetes mellitus (DM) with repaglinide and dimethyldiguanide treatment, and obstructive sleep apnoea-hypopnea syndrome treated by nasal septal reconstruction. He had never smoked or drank, was exposed to live poultry or travelled.

His pulse was 122 beats per minute; his respiratory rate was 35 breaths per minute; and his blood pressure was 148/86 mmHg. Auscultation revealed rales in the bilateral basic lung fields. Arterial blood gas analysis showed respiratory alkalosis and respiratory failure type I (pH 7.49; pCO_2_: 29 mmHg; pO_2_: 43 mmHg; HCO_3_^−^: 22.1 mmol/l).

**Fig. 1:**
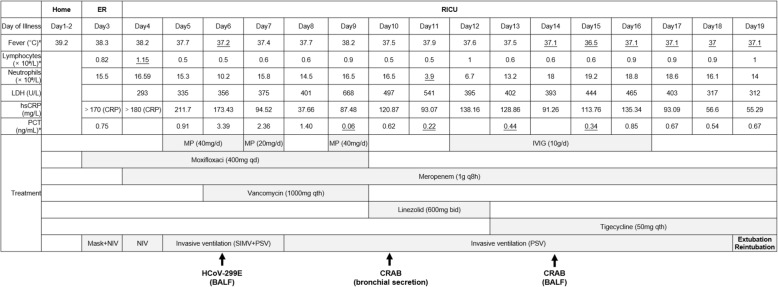
Time schedule of clinic course and laboratory parameters based on days from symptom onset, from Nov 2 to Nov 20, 2019. *The normal values of parameters were underlined in the figure. BALF, bronchoalveolar lavage fluid; CRAB, carbapenem-resistant *Acinetobacter baumannii;* ER, emergency room; HCoV-229E: human coronavirus 229E; hsCRP, high sensitive C-reactive protein; IVIG, intravenous immunoglobulin; LDH, lactate dehydrogenase; MP, methylprednisolone; NIV, non-invasive ventilation; PCT, procalcitonin; PSV, pressure support ventilation; RICU, respiratory intensive care unit; SIMV, synchronized intermittent mandatory ventilation

**Fig. 2:**
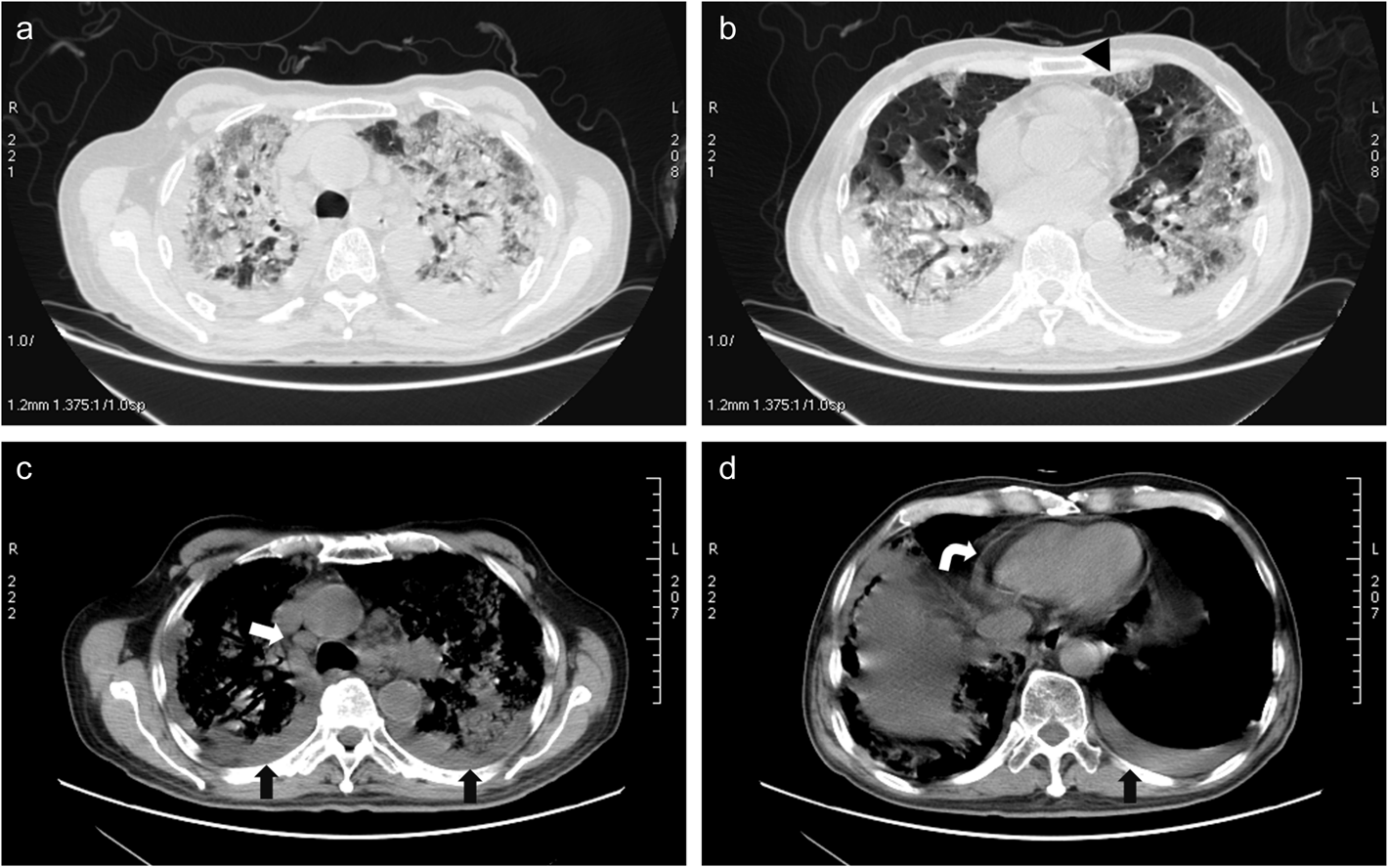
Non-contrast enhanced thin-section CT scans on day 4 after symptom onset. **a**, **b** Lung window, bilateral ground-glass opacities with clear boundary, multifocal consolidations, air bronchograms, smooth interlobular and intralobular septal thickening could be seen (black triangle). **c**, **d** Mediastinum window, lymphadenopathy (white arrow), bilateral pleural effusions (black arrows), and moderate pericardial effusions (curved white arrow) were shown

Laboratory analyses (Fig. 1) revealed lymphopenia, neutrophilia, high C-reactive protein (CRP) levels and elevated lactate dehydrogenase (LDH) levels. Levels of brain natriuretic peptide (248 pg/ml) and cardiac troponin I (0.042 ng/ml) slightly increased. Levels of glycosidic hemoglobin (7.5%, normal range: 4.0 ~ 6.0%), ferritin (729 ng/ml, normal range: 23.9 ~ 336.2 ng/ml) and Interleukin-6 (10.63 pg/ml, normal range:<6.4 pg/ml) increased. D-dimer level on admission were above the normal range (3.65 mg/l), and color doppler ultrasound revealed soleal and gastrocnemius vein thrombosis in both legs. Chest computed tomography (CT) showed diffuse ground-glass opacities (GGOs) with consolidation, pleural effusion, pericardial effusion and lymphadenopathy (Fig. 2). Intravenous moxifloxacin and noninvasive ventilation were given.

He was transferred to the respiratory intensive care unit (RICU, day 4) and received invasive ventilation (day 5) due to rapidly progressing to acute respiratory distress syndrome (ARDS). Fibreoptic bronchoscopy was performed (day 6), the bronchoalveolar lavage fluid (BALF) was bloody with an increased proportion of neutrophils (89%) and eosinophils (2%). Metagenomics next-generation sequencing (mNGS) of BALF found the only pathogen of HCoV-229E (sequence reads: 5195 bp). Culture and smear of lower respiratory tract secretions were negative for bacteria and fungus. Nucleic acid tests for common respiratory virus, mycoplasma and chlamydia were negative. Pleural fluid was sterile transudate dominated by mononuclear cells (62%). Procalcitonin (PCT) increased significantly after endotracheal intubation. Short-term and low-dose methylprednisolone and intravenous immunoglobulin (IVIG) were given. Low molecular weight heparin was given for deep venous thrombosis (DVT). Broad spectrum antibiotics were empirically used (Fig. 1).

On day 8, paroxysmal atrial fibrillation occurred without cardiac structural changes. On day 14, repeated bronchoscopy was performed for increased infiltration in the left upper lobe. Cytological analysis of BALF showed an increased ratio of neutrophils (93%). Culture of BALF was positive for carbapenem-resistant *Acinetobacter baumannii*, and antibiotics were administered.

**Fig. 3:**
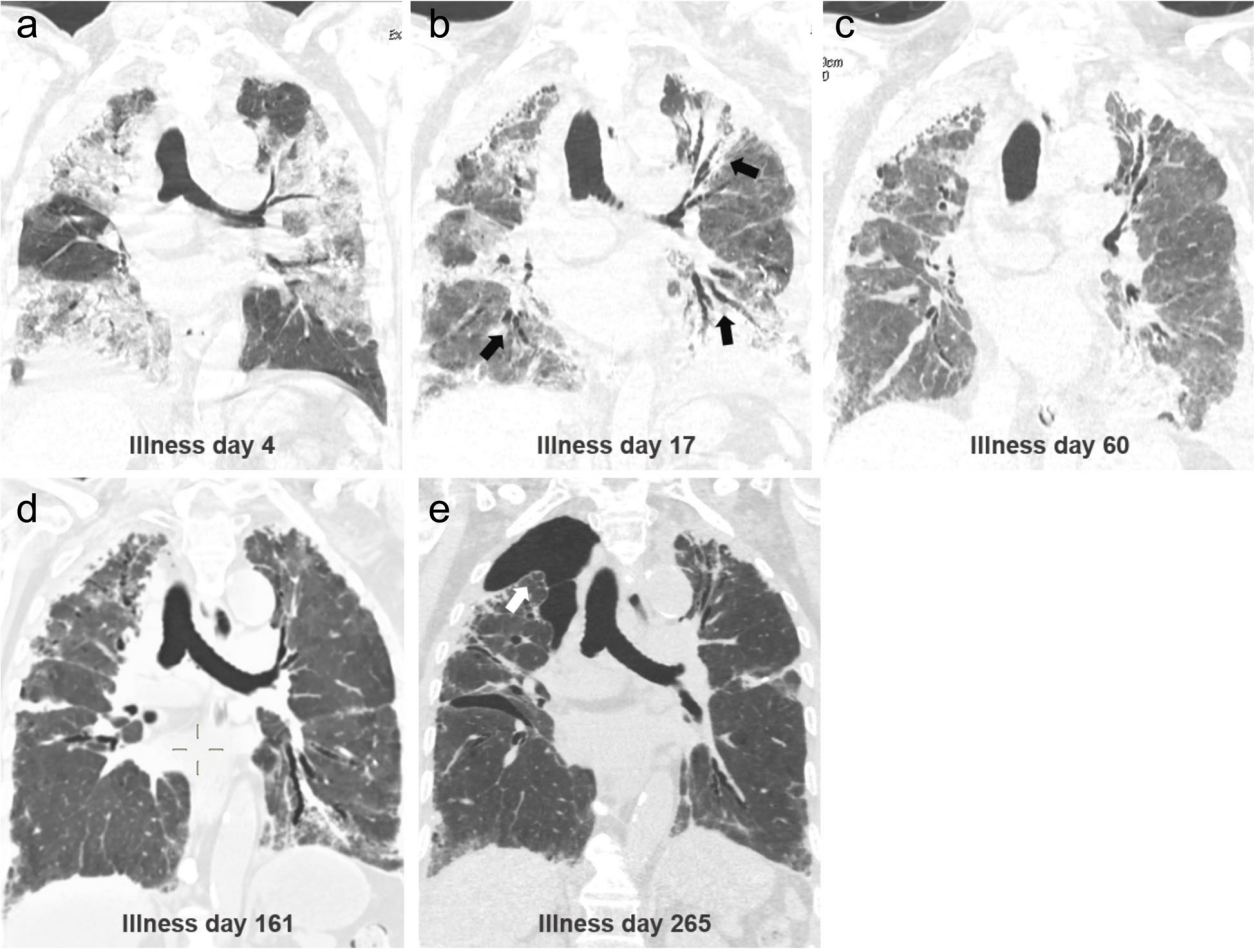
Non-contrast enhanced coronal-reconstruction CT scans on day 4, day 17, day 60, day 161 and day 265 after symptom onset. **a** Bilateral diffuse ground-glass opacities with consolidations. **b** Lung volume reduced associated with reticular and traction bronchiectasis (black arrows) in both lungs and consolidation in the left lower lobe. **c** Absorption of bilateral pulmonary lesions, focal honeycomb lung in the right upper lobe, coupled with traction bronchiectasis in the left upper lobe. **d** Further resolution of lesions remaining fewer fibrosis changes in the bilateral lung field. **e** Absorption of lung fibrosis with a focal pneumothorax in right upper lobe (white arrows)

On day 19, the patient fulfilled the criteria of withdrawal from mechanical ventilation but failed, due to ICU acquired weakness (ICUAW) and persistent atrial fibrillation. After reintubation, he suffered from ventilator-associated pneumonia. Bacterial culture of lower respiratory tract secretion yielded positive for carbapenem-resistant Klebsiella and methicillin-resistant *Staphylococcus aureus*. Avibactam-ceftazidime, teicoplanin, or linezolid was successively used, together with a conservative liquid strategy, limb rehabilitation exercise, phrenic nerve stimulation, and nutrition support under intensive glucose control. The patient was successfully weaned from ventilation on day 74 and discharged from the hospital on day 105. He remains well during the regular follow-up. Radiographic findings (Fig. 3) showed the development and gradual absorption of fibrosis and recent focal pneumothorax in right upper lobe.

**Fig. 4:**
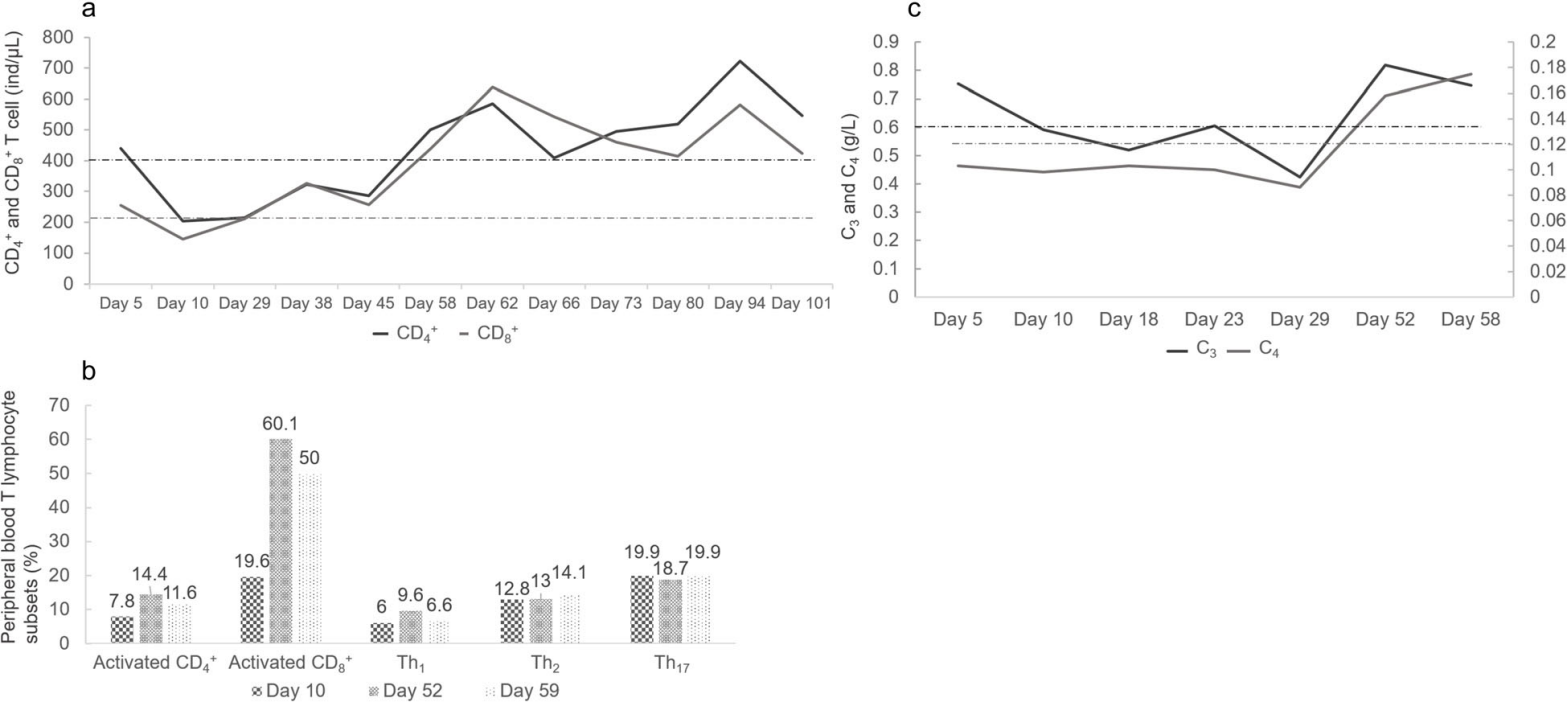
The changes of peripheral T cells and complements. **a** The changing trend graph of the count of CD_4_^+^ and CD_8_^+^ T cells in peripheral blood. The low limit of normal value (CD_4_^+^ T cell, 404 cells/mm^3^; CD_8_^+^ T cell, 220 cells/mm^3^) was shown by a dashed line. **b** Changes of peripheral blood T lymphocyte subsets. The normal range of activated CD_4_^+^ T cells was 2.4% ~ 9.6%, of activated CD_8_^+^ T cells was 3.8% ~ 32.4%, of Th_1_ cells was 0.6% ~ 19.0%, of Th_2_ cells was 0.3% ~ 2.7%, and of Th_17_ cells was 10.8% ~ 39.0%. **c** The changing trend graph of serum complements. The low limit of normal value (C_3_, 0.6 g/L; C_4_, 0.12 g/L) was shown by a dashed line

The absolute counts of lymphocyte subsets were measured by flow cytometry in peripheral blood (Fig. 4). The absolute number of CD_4_^+^ and CD_8_^+^ T cells decreased within 1 week and recovered after 45 days, as well as the levels of complements showed the same trend. There were no changes in the proportion of T-helper 1 (Th_1_) and Th_17_ cells. The proportion of activated CD_4_^+^ and CD_8_^+^ T cells began to increase after day 10. The proportion of Th_2_ cells persistently increased from day 10 and lasted for more than 40 days.

## Discussion and conclusions

HCoV-229E is one of four non-severe acute respiratory syndromes (SARS)-like CoVs (HCoV-NL63, HCoV-229E, HCoV-OC43, and HCoV-HKU1) causing the common cold, and severe lower respiratory infection caused by HCoV-229E is predominantly found in the elderly, children under 2 years of age, and those with immunosuppression from any cause [3–5]. DM was associated with a higher rate of mortality and morbidity in non-SARS-like CoV (HCoV-NL63), and SARS-like CoV (SARS and COVID-19) [6–8]. Hence, we speculated that old age and DM might play a crucial role in the severity of infection in this case.

The prevalence of non-SARS-like CoV-associated ARDS was extremely low in adult (0.1%) [9]. In this case, disease progression in the first 7 days was mainly caused by HCoV-229E because it was the only pathogen, and antibiotic therapy was ineffective. The median duration to ARDS was 4 days, which was quicker than that of COVID-19 [6]. Similar to COVID-19, this patient had all of the factors of a poor prognosis including lymphopenia, neutrophilia, elevated LDH levels, high CRP levels, increased D-dimer and abnormal myocardial indices [6]. Moreover, new onset atrial fibrillation and ICUAW, which were mostly identified in systemic inflammatory response syndrome, might have contributed to the prolonged hospital stay of our patient than that of HCoV-infected patients in a previously long-term study (average, 12 days; range, 5–30 days) [8].

To our knowledge, no researches were available on the continuous image in patients with HCoV-229E pneumonia. The CT chest imaging of our patient manifested as GGOs and consolidations in multiple lobes throughout both lungs, which were the predominant features within 2 weeks, similar to COVID-19 [10]. In addition, mediastinal lymphadenopathy, pleural effusion and pericardial effusion of our patient, which were rare in COVID-19, might be related to viral-induced myocardial damage and exuberant inflammatory response [10, 11]. Elderly patients and those with critical cases of SARS were likely to develop fibrosis [10, 12]. In our case, fibrosis developed early (day 17), absorbed gradually, and lasted for at least 8 months.

There are no data on the specific role of either humoral or cellular immunity or innate immunity in patients recovering from HCoV-229E. Similar to COVID-19 [6, 13], the initial decrease in the number of lymphocytes and CD_4_^+^ and CD_8_^+^ T cells in this case might suggest a dysregulated cellular immune response [14]. Low levels of complements were reported to be a risk factor for high mortality in SARS [15]. The proportion of Th_17_ cells was persistently normal in this patient, albeit with an increased concentration of highly proinflammatory CCR_4_^+^CCR_6_^+^Th_17_ cells in COVID-19 [13]. The time to immunological recovery of this case was 2 months when the patient was liable to acquire secondary infections. Thus, we suggest that patients with severe HCoV infection should be closely monitored for over 2 months.

Whether HCoV infection could benefit from corticosteroid therapy has remained controversial. It was illustrated that early corticosteroid treatment could induce higher viral loads and delayed viral clearance [16]. However, recent studies from COVID-19 revealed that the use of corticosteroids was associated with shorter hospitalization stays [17], and reduced 28-day all-cause mortality among critical patients with receiving respiratory support [18, 19]. This case highlighted that low-dose and short-term corticosteroids at an early stage might reduce the excessive inflammatory response.

Currently, there is no definitely effective antivirals for the treatment of coronavirus infection. Efficacy of IVIG for severe HCoV infection needs further evaluation. Experience from our patient emphasized the importance of supportive care strategies as provided in previous recommendations [20], including timely mechanical ventilation, intensive glucose control, rehabilitation, treatment of DVT and secondary infection control.

There were several limitations. First, we lacked detection technology for virus titres. Second, we were not sure when the virus was completely cleared, as the family refused repeated mNGS tests. Third, we did not test the T lymphocyte subset within 1 week.

In conclusion, the case of severe HCoV-229E pneumonia with poor prognostic features indicated the necessity of closely monitoring multiorgan involvement associated with HCoV infection. Suboptimal T cell response and excessive consumption of complement would last for 2 months in severe cases, contributing to a high risk of secondary infection. Supportive therapy should be indispensable for HCoV-229E infection.

## Data Availability

All the information generated or analyzed during this study are included in the manuscript. There are no datasets related to this case report.

## Abbreviations

ARDS: Acute respiratory distress syndrome
BALF: Bronchoalveolar lavage fluid
COVID-19: Coronavirus disease 2019
CRAB: Carbapenem-resistant Acinetobacter baumannii
DM: Diabetes mellitus
HCoV: Human coronaviruses
CRP: C-reactive protein
CT: Computed tomography
GGOs: Ground-glass opacities
ICUAW: Intensive care unit acquired weakness
IVIG: Intravenous immunoglobulin
LDH: Lactate dehydrogenase
mNGS: Metagenomics next-generation sequencing
PCT: Procalcitonin
RICU: Respiratory intensive care unit
SARS: Severe acute respiratory syndrome
Th: T-helper
